# A measure of social coordination and group signaling in the wild

**DOI:** 10.1017/ehs.2020.24

**Published:** 2020-05-14

**Authors:** Adrian Viliami Bell

**Affiliations:** Department of Anthropology, University of Utah, Salt Lake City, UT 84112, USA

**Keywords:** Coordination, ethnic markers, signals, classification, Tonga, Polynesia

## Abstract

Adaptive interactions in large populations often require honest signals of group membership to structure interactions. However, limitations to a simple mapping of groups onto stylistic and ethnosomatic variation suggest that new ways of measurement are needed to describe the work that objects do to facilitate social coordination. Means to measure the benefits to coordinating on specific objects, here called signaling value, would transition inquiry from general statement that signals play a role, to which signals play what roles in what contexts. This study introduces a method to measure the signaling value of specific objects using classification tasks. After mathematically showing how social coordination leads to greater associations in object classification, a statistical approach is derived to estimate the signaling value of objects from a triad classification task. The approach is then applied to a study of culturally salient motifs in the Pacific Island nation of Tonga and a comparison group in the US. The statistical estimates suggest a large role for social coordination for the full set of motifs, although there is a substantial range of signaling values among motifs. In light of the estimates, the cultural history of individual motifs is discussed as well as the future of this approach.

## Graphical abstract

**Figure:**
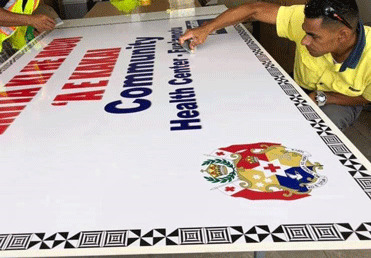
Production of a sign for a new health center in the Kingdom of Tonga. Note the cultural significant motifs, or *kupesi*, comprising the border.

**Media summary:** Whether we realize it or not, all of us use dress, language, body markings or other outward signs to communicate our ethnic, religious or other group affiliations. If we can decipher the role of specific signals, then we can understand more deeply how human societies organize themselves to coordinate, cooperate and maintain aspects of culture. However because individuals have multiple affiliations that change over time, it is difficult to rank the importance of specific signals. This article addresses this problem with a new approach. By measuring how individuals classify a set of candidate signals, it shows how to estimate the value of each signal. This method is applied to a study of motifs in the Kingdom of Tonga.

One of the solutions to the question of large-scale human adaptability includes the structuring of human expression to promote social coordination (Goffman, 1978; Skyrms, 2004). Objects or signals are often the focus of successful coordination efforts (Cohen, 2012; Nettle & Dunbar, 1997; McElreath, Boyd, & Richerson, 2003), which comprise the ‘social facts’ and norms that make up key features of social identity and the boundaries between groups (Durkheim, 1982; Wimmer, 2013). However, despite its central place in thinking of human behavior, a measure of an object's value to coordination has not been attempted, preventing vital assessments of the theories behind the evolution of group signals. This paper reports a quantitative attempt at filling this key gap through a theoretical and statistical investigation of objects expressed in an ethnographic setting. Using the methods of object classification, it is shown how the way individuals classify objects can allow us to estimate an object's signaling value for social coordination.

In large populations social coordination is a formidable problem given the segmented and nested structure of group identities. Multiple ethnolinguistic, political and wealth classes are present with individuals navigating the social milieu to gain benefits with in-group members (Cohen, 2012) and inform their interaction with out-group others (Goffman, 1978). The solution may be through ethnosomatic traits, although this solution is restricted to conditions where behavioral and cultural variation is limited among those of relatively close genealogical descent. This is an unlikely scenario for migrating populations whose socioecological adaptations contrast with their origin cultures (Portes & Rumbaut, 2014). Further, social network studies in multiethnic populations show that, while racial and ethnic homogeneity is common (McPherson, Smith-Lovin, & Cook, 2001), a sophisticated model of network formation downplays the effects of homophily owing to the equally strong effects of reciprocity and propinquity (Wimmer & Lewis, 2010). Thus bodily features including color and shape will often be poor predictors and organizers of those with shared interests.

Famously it was argued that stylistic variation may effectively signal the presence of group boundaries (Barth, 1969; Hodder, 1982). However, signals without much cost beg the question of signals evolving without strong enough correlations to the social norms they are presumed to transmit (Grafen, 1990). Theoretical clarification corroborated that honest signals can evolve to signal group membership under certain conditions (Boyd & Richerson, 1987; McElreath *et al.*, 2003). Framed around solving coordination games, these models show that signals may highly covary with behavior, especially in ethnically diverse contexts, where migration rates are moderate, and there are high enough benefits to social coordination. The empirical tests of the theory since then come from experimental work (Efferson, Lalive, & Fehr, 2008) and gathering new and published accounts of how individuals navigate multiethnic spaces (Goffman, 1978; Moya & Boyd, 2016; Wallman, 1986). However, while experimental work has shown signals to evolve as predictors of behavior (Efferson *et al.*, 2008), outside the laboratory or in ‘the wild’, a statistical measure of an object's role in social coordination has been elusive.

One major obstacle is that ethnographic studies challenge any simple assigning of objects to groups. In a number of studies there is mixed evidence for clear mapping of stylistic variation on ethnolinguistic groups (Hodder, 1982; Wiessner, 1984). A vignette study aiming to estimate visual predictors of latent beliefs or behaviors showed high variability in supported traits between age classes in an urban and agropastoral population (Moya & Boyd, 2016). This pattern of loose ethnic marker mapping continues with a 2016 study of Tongan motifs led by the author, where the motifs showed high variability in their self-reported knowledge and use (Bell and Paegle, unpublished observations). By showing that individuals have different recognition levels for motifs produced by their own ethnolinguistic group, it follows that some motifs probably have a higher social coordination value than others. Further, variation is introduced spatiotemporally as globalization and migration separates ethnic identity with previously prevalent cultural norms (Bell, 2012; Morton, 1998). In sum, a general feature emerges that candidate group signals are easy to nominate but hard to evaluate what work they actually do to demarcate groups.

## Relationship between social coordination, signals, and classification

One solution to deciphering the relevance of candidate signals lies in methods describing the construction of categories. Often used to measure a population's response to social, cultural and environmental conditions, object classification tasks are an ethnographic method that describe how populations organize what they observe (Weller, 2015). The promise is that how a society organizes objects is in part due to what concerns the individual – food, the weather and the need to coordinate with others. An empirical study of how a population organizes objects can then be used to reconstruct the details of social coordination.

To see how this might work, let us imagine a population of individuals that play coordination games on three objects, {1, 2, 3} – the smallest possible number of objects in a classification scheme. Each individual associates two objects together, either {1,2}, {2,3} or {1,3}, and interacts with others in the population, gaining benefits depending on how many objects match their partner's. Two individuals both with {1,2} gain more benefits than two individuals having {1,2} and {1,3}, given that the former has two objects in common and the latter only one. Further let us also say that some objects lead to greater coordination payoffs than others, if the coordination payoff to object 2 is greater than that to object 3, such that two individuals coordinating on {1,2} have greater payoffs than when they coordinate on {1,3}. Assuming that differential reproduction of the object pairs is an increasing function of these payoffs, then we can solve for conditions when two objects are associated more together than alternatives.

Mathematically, if *δ*_*i*_ is the benefit acquired from two individuals coordinating on object *i*, hereafter called the signaling value, and *x*_*ij*_ is the frequency of two objects *i* and *j* associated together, then the condition for the pair {1,2} to increase in frequency is:1
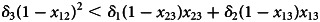


**Figure 1.**
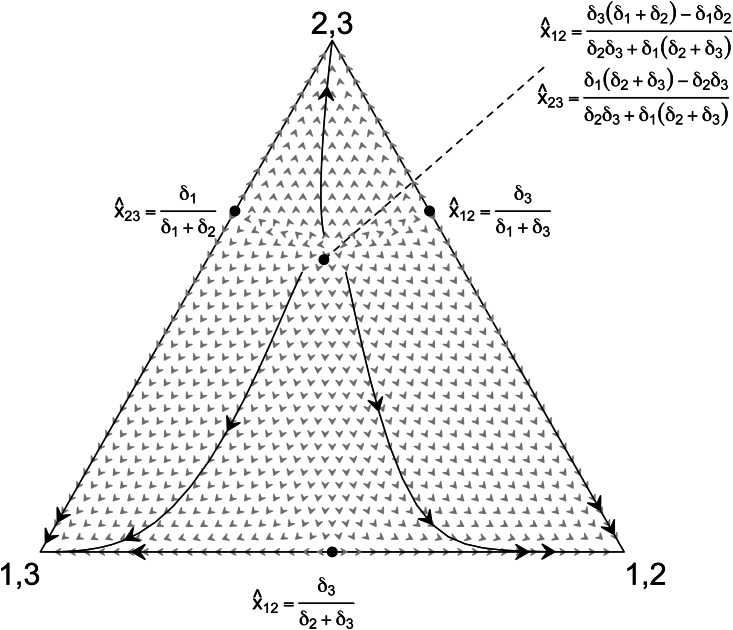
Coordination benefits on single objects affect the association between objects. A ternary plot phase diagram is shown with points marking unstable equilibria for a system of difference equations linking social coordination and object association. Note the labeled unstable equilibria and hence the scope for associations {1,2}, {2,3}, and {1,3} are functions of signaling values (*δ*_*i*_). Arrows point to the direction of the system. Signaling values used to plot the system are *δ*_1_ = 1, *δ*_2_ = 0.5, and *δ*_3_ = 0.5. See Supplementary Material for model details.

This expression says that the signaling value to object 3 must be less than a linear combination of the signaling values to objects 1 and 2 scaled by the frequency and/or variance of relevant pairs (see Supplementary Materials). In other words, two objects may be classified together if at least one has a higher signaling value and high enough frequency in the population. This relationship is explored in detail in Figure 1.

The link between signaling values and object pairing as demonstrated by this model provides an opportunity. If object classification reflects socialization through coordination benefits, we can reverse-engineer object classification data to measure the signaling value (*δ*_*i*_) for each object and the extent of social influence on the whole set. The statistical methods below develop this approach, which is then applied to calculating the signaling value of six culturally salient motifs among the Tongan ethnolinguistic group.

Each motif is produced within and population and motifs generally are known to be key representations of Tongan identity. However, each motif is very likely to have varying social significance as suggested by previous ethnographic work (Bell and Paegle, unpublished observations). This motivates the social coordination approach that aims to isolate the differential importance of each motif in the population. Thus, with triad classification data collected by the author's research team in the Tongan islands and in a contrasting US population, model parameters were estimated describing the signaling value of the motifs as well as the degree of social influence on the whole motif set.

## Materials and methods

Following the motivating model in the Introduction, we aim to statistically estimate the requisite level of social coordination that may explain how individuals classify objects. To do this we take a modeling approach that represents both inherent classifications between objects and social coordination, with the latter the theoretical focus of this study. The inherent classification component reflects the nature of classification tasks and some social scenarios where a response is required (forced choice) even if the person knows nothing of the objects. The consequence is that individuals will group objects by features that have no connection to social coordination, e.g. stylistic similarities.

To measure the classification of a set of objects *A* = {1, 2, …, *N*_*A*_} a common approach is to take a subset *B* of three objects from *A* (*B*⊆ *A*) and ask a respondent to pair two objects as most similar by choosing the most dissimilar object (Weller, 2015). Called the triad classification task, this method is least costly to implement in field data collection. Once all combinations of three are completed, a classification measure among all objects is complete. The model below is designed for this type of classification data.

For any given subset *B*, to predict whether a pair of objects is classified together given a the set of three, 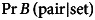, we will weigh the two plausible causal forces of social coordination and inherent classification with the parameter *u*:2
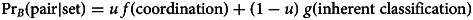


where higher values of *u*, from zero to one, indicate the greater use of social coordination. We now specify the functions *f* and *g* that capture social coordination and inherent classification, respectively.

Inherent classification may be estimated using data collected in populations previously not exposed to the objects. We can estimate the probability of two objects, e.g. {1, 2} out of three {1, 2, 3} associated together, *g*(1, 2|1, 2, 3), by the fraction of pairs made in the triad classification task across *N*_*u*_ individuals in this population. This follows from the multinomial probability function, where the fraction of the time a pair is chosen empirically is equal to the maximum likelihood estimate of its probability of being chosen in a multinomial probability model. Thus,3
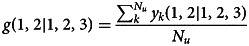


where across all *N*_*u*_ individuals indexed by *k* in the unexposed population, *y*_*k*_(1, 2, |1, 2, 3) = 1 if the pair {1, 2} is grouped together, zero otherwise. This can be computed for any pair within a triad.

The classification of objects reflects in part the benefits received owing to social coordination, represented by the function *f*. Table 1 details a model of interaction and the spread or decline of object pairings *x*_*ij*_ owing to payoffs in a coordination game. As in the theoretical model above (equation (1)), parameter *δ*_*i*_ denotes payoffs to coordinating on one object *i* such that coordinating on one object, say *δ*_1_, may be higher than coordinating on another object, say *δ*_2_. The strength of selection is scaled by parameter *β*_*S*_.

**Table 1.** Table outlining the outcomes of coordination games played between individuals *K*_1_ and *K*_2_ expressing object subset *B* out of set *A* = {1, 2, 3}. 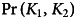 is the probability that two individuals with pairs *K*_1_ and *K*_2_ interact, and 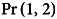, 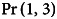 and 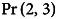 are the probabilities that pairs {1, 2}, {1, 3} and {2, 3} are chosen as a result of the interaction, respectively. Parameter *δ*_*i*_ denotes payoffs to coordinating on object *i* in the set while *β*_*S*_ scales the strength of selection. The frequency of an object combination *i* and *j* is *x*_*ij*_. The function *H* is the inverse-logit function *H*(*x*) = *e^x^*/(1 + *e^x^*), allowing for any row that 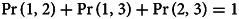 and 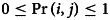 for all Real values potentially taken by parameters above. This ensures a continuous likelihood surface for effective sampling of the parameter space by estimation methods

*K*_1_	*K*_2_	Pr(*K*_1_, *K*_2_)	Pr(1, 2)	Pr(1, 3)	Pr(2, 3)
12	23	2*x*_12_*x*_23_	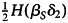	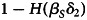	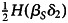
12	13	2*x*_12_*x*_13_	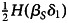	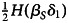	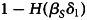
23	13	2*x*_23_*x*_13_	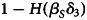	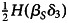	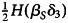
12	12	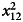	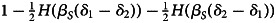	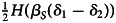	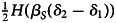
23	23	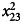	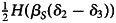	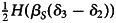	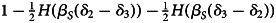
13	13	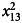	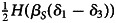	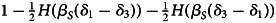	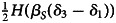

From Table 1 we derive a recursion tracking the frequency of each object combination through time (see Supplementary Materials). This is done by summing the multiplied columns of Pr(*K*_1_, *K*_2_) and Pr(*i*, *j*) for any pair {*i*, *j*} to give its frequency in the next generation. By simulating the frequency of *x*_12_, *x*_13_ and *x*_23_ to equilibrium, we explore the long-run expression of each pair of objects as a function of signaling value (*δ*_*i*_).

**Figure 2.**
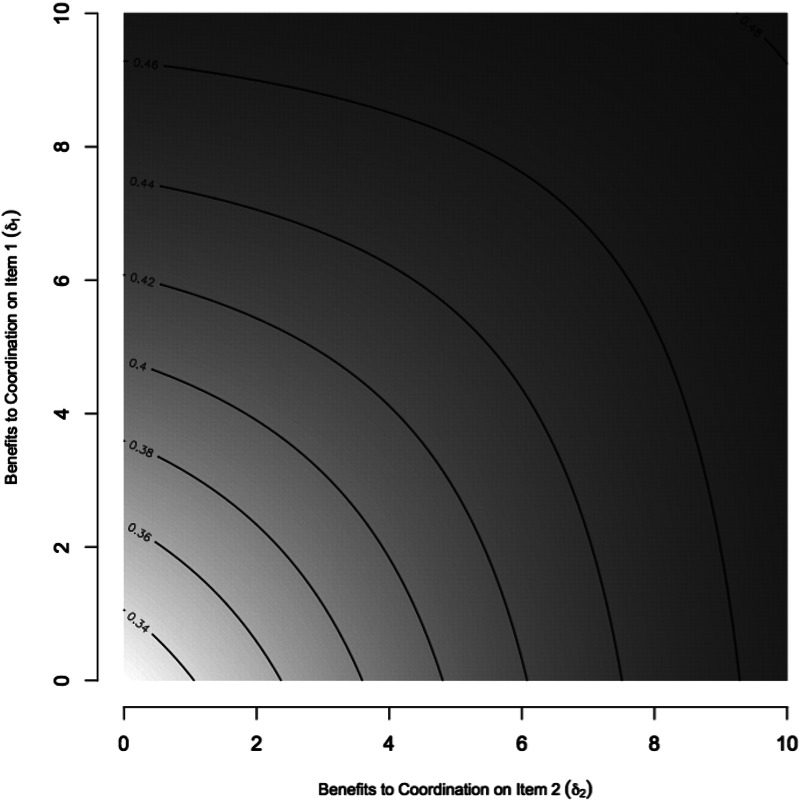
Properties of the recursion generated by Table 1. Plot of the equilibrium frequency of object pair {1, 2} as a function of the coordination benefits to item 1 (*δ*_1_) and item 2 (*δ*_2_). With darker regions corresponding to higher frequencies, the plots show how the integrative benefits to coordinating on one or multiple objects make the grouping of the two more likely in the long run. Parameters set as constant are: *δ*_3_ = 0.3 and *β*_*S*_ = 0.3.

We explore some features of the dynamics (Figure 2), confirming the positive relationship between coordination benefits between objects and their joint classification (equation (1)). Figure 2 shows that two objects may be more consistently classified together if at least one has a high signaling value. This equilibrium frequency is used for a specific pair of objects (e.g. *x*_12_) as a predicted probability that a pair of objects is grouped together from a set of three, constructing *f*(coordination) in equation (2). That is, 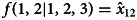.

With *f* and *g* defined we can find the empirical likelihood. Given a subset of objects, for example *B* = {1, 2, 3}, and the parameter set *θ* = {*u*, *β*_*S*_, *δ*_1_, *δ*_2_, *δ*_3_}, the probability that a particular pair is chosen by a participant *k* is
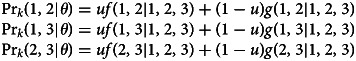


It follows that the probability of a particular choice made by participant *k* for subset *B* = {1, 2, 3} is
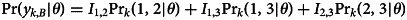


where *I*_*i*,*j*_ is an indicator function with value 1 if the pair {*i*, *j*} is grouped by individual *k*, zero otherwise. The likelihood is thus,4
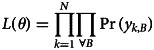


which we use to find maximum likelihood estimates 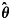.

### Ethnographic methods

In the Kingdom of Tonga in the South Pacific, native peoples often manufacture a set of motifs or *kupesi* that are found within the island and in the Tongan diaspora. Many of the motifs have names and are taught in Tongan schools, and the manufacture of motifs is a cultural and economic activity tied to important life events in the public sphere. As in other parts of the Pacific (Ewins, 2004), the motifs are seen as key representations of social concepts and identity, and thus key candidates as honest signals of Tongan group membership. However despite their presumed importance, previous ethnographic work led by the author documented high variability in the recognition and naming of motifs (Bell and Paegle, unpublished observations), suggesting that the signaling value of each motif is also variable. Thus these disputed motifs provide an good test case for applying the statistical methods described above.

In 2018 a team led by the author conducted ethnographic surveys in Tonga. As part of the survey, 114 participants completed 20 triad classification tasks for six cultural significant motifs selected from previous work (Bell and Paegle, unpublished observations). The tasks were administered in homes, at markets and on the street to 56 women and 55 men, ranging in age from 18 to 70, on a touchscreen tablet. In order to estimate the effect of inherent classification (3), the triad classification task was conducted with 51 University of Utah students who had no prior exposure to the motifs, although in English.

**Figure 3.**
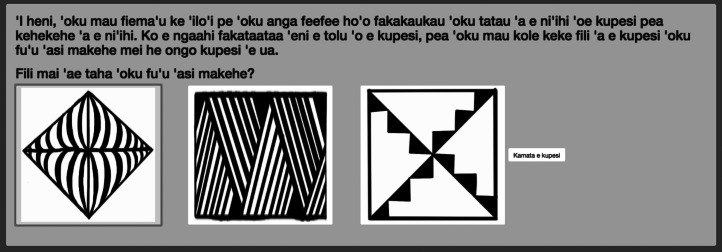
Screen shot of the instructional prompt of the triad classification task in Tongan. Administered on a touch-screen tablet, individuals are instructed to choose the motif most different from the other two. After each choice another set appears continuing until all triads of six motifs have been completed, or 20 decisions. In this particular shot, an individual chose the first motif on the far left.

In both populations, the potential participant was asked for consent to participate in a study about culture and was directed to the instructional screen shown in Figure 3. This screen also served as practice with the touchscreen interface. To ensure that the classification tasks represented motif organization and possibly social coordination rather than exposure, six well-known motifs were chosen. The order of the motif triads presented on the screen was randomized across individuals. The touchscreen survey was coded by the author using R's web interface Shiny R and run locally. A cleaned version of the data and analysis is deposited on the author's GitHub account (avbell/SignalingValue). All activities were approved beforehand by the University of Utah ethics board (IRB #00050947).

#### Model fitting

In the statistical program R we computed the likelihood (4) and used numerical optimization methods to find maximum likelihood estimates. An investigation of the likelihood surface shows a smooth surface (Supplementary Figure S2) and simulations were conducted to show that the estimation routine yielded unbiased estimates of the data-generating parameters (Supplementary Figure S1). Because computing confidence intervals via the Hessian often resulted in an incomplete variance–covariance matrix, the parameter distributions were estimated using Markov-Chain Monte Carlo vis Metropolis–Hastings random sampling (Supplementary Figure S2). The data and routine in R are deposited in GitHub (see Supplementary Materials for details).

## Results and discussion

**Figure 4.**
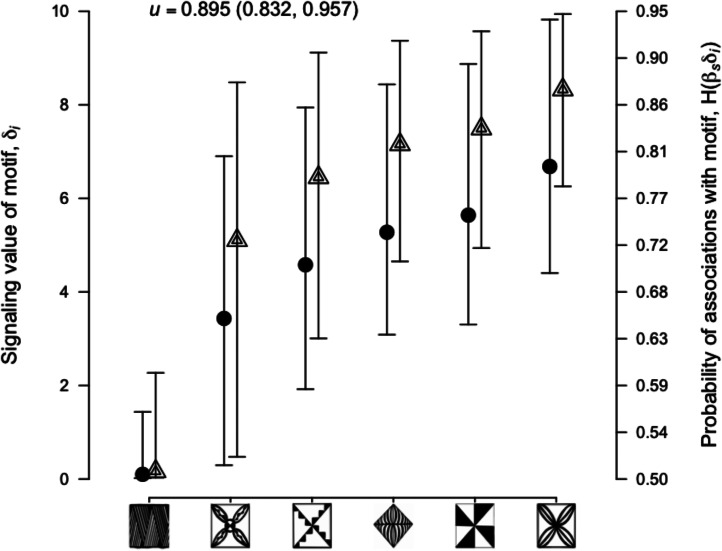
Model estimates. Plotted on the left axis with the circles is *δ*_*i*_, the signaling value of an object *i*. The right axis with triangles is the probability increase for association with a motif *i*, or 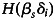 (see Table 1). Parameter distributions were calculated using a random walk Metropolis–Hastings sampling of the likelihood, with mean, 2.5% and 97.5% quantiles shown. For effective sampling, the strength of selection coefficient was set at *β*_*S*_ = 0.286.

The primary question of social influence was answered clearly. The parameter weighing the effects of social coordination with inherent classification, *u*, was estimated to be 0.895 with satisfactory precision (Figure 4). This suggests that the majority of the classification data can be explained by in-group social learning and signaling processes rather than an out-group classification of motif designs that revolves around stylistic similarities – circles grouped together with circles and squares with squares. The estimates of individual motifs are also revealing.

With the signaling values ordered in Figure 4, we see a gradual increase in importance across motifs with the exception of one motif with a very low estimate (Figure S2). The larger signaling values also signify greater associations made with the motif, as shown by the mathematical model (Figure 1). At the high end, associations with the motif to the far right will be made around 88% of the time, the next motif around 84%, then 82% and so on down to a random coin flip for the far left motif at 50% (see Table 1). Taken together, the estimated values suggest that while the whole set is under social influence, some motifs play greater roles in social life than others. In an evolutionary scenario, the motifs with greater signaling values would eventually become more prominent. While no separate test is undertaken here to evaluate this result, an appeal to culture history may illuminate possibilities.

The estimated signaling values may reflect their documented cultural–historical significance. The highest value was given to a motif found on the earliest Lapita pottery associated with Austronesian speaking peoples out of the South-western Pacific over 3000 years BP (Cowling, 2009; Spriggs, 1990) who eventually settled Tonga. A variant of the motif called *manulua* in Samoa and Tonga, it is adapted today to reflect a fondness for flowers by residents and tourists, especially the flowering plants from the genus *Plumeria* (Cowling, 2009). This motif's reproduction is found in every conceivable medium both traditional and modern in Tonga and Samoa. Following this motif is the most popular variant of *manulua* of more triangular forms (Helu, 1999). While many motif variants may be the result of local innovations, some argue that this *kupesi* represents the four wings of two birds marking the joining of chiefly lineages. Like much of Pacific artwork that contains both naturalistic and abstract symbols, these ‘named *kupesi*’ convey the Tongan concept of *heliaki* – to say one thing but mean another. The next elongated diamond-shaped motif is known in Tonga as *tokelau feletoa*; while attributed to the island group of Vava'u within Tonga, it is seen throughout the Tongan archipelago. While the form of *tokelau feletoa* has multiple interpretations (Arbeit, 1994; Helu, 1999), they all refer to chiefly symbols.

What of the motif with the lowest signal value? Called *amoamokofe* or literally ‘inner plant bark with bamboo’, the straight line designs reflect an older tradition of high abstraction, angularity and extreme simplicity (Helu, 1999). While ancient like the oldest *manulua*, it probably lacks strong appeal compared with other modern objects that, whether in the eyes of visitors or native peoples, represent Tongan and more broadly Polynesian identity today. Modern *kupesi* that represent today's chiefly class have motifs with higher degrees of realism – a coat of arms, lion or eagle, or a row of Norfolk pines (*Hala Paini*) (Helu, 1999).

### Future work

While model estimates suggest that the set as a whole is under strong social influence, the signaling values are relative to others in the set and thus inferences are limited by the set of objects classified. It is key, therefore, to identify salient objects and understand their contextual limitations. As the theoretical and statistical models introduced here assume that the signaling values are stable attributes of the population, the analysis should be applied with a comparative approach that captures the spatiotemporal dynamics of signals.

Thus while the motifs studied here have undoubted ethnographic importance among Pacific cultures (Ewins, 2004; Helu, 1999), future work should consider variations on older motifs and the creation of entirely new ones as innovations in Tonga and in the Tongan diaspora introduce a new suite of potential signals among the next generation. Thus object sets in future study may solely include older objects, only include modern innovations or contrast older objects with more modern innovations. In addition, some objects may be added as obvious outliers – flags, national colors, out-group symbols – to create a baseline for other objects. By varying the object sets across contrasting groups, one may test for group signaling across specific spatial and/or temporal dimensions.

Because it is possible that stylistic similarities between motifs are caused by their coordinated signaling value, then this paper might underestimate the social coordination value of the motif set. This follows from the choices of the unexposed reference population, which if they classify based on stylistic similarity, will cause the model in (2) to attribute classifications to the reference group rather than social coordination in the target population. Given that some of the motifs shown in Figure 4 appear more stylistically similar, there is the possibility that *u* is underestimated. This may be solved by choosing an object set that reduces redundancy by eliminating motifs that may be playing the same social role.

There are probably better data collection protocols to measure signaling value. For example, one may devise various prompts, from the more conservative and ambiguous ‘which object is most different?’ used in this study to the more directed ‘which object doesn't belong here?’ The latter may elicit stronger social categorization of objects than what is found in this study. Also, other variations of the classification task and object clustering may be used as an ethnographic method, e.g. pile sorts. The clustering approach may then allow social value to be estimated for an arbitrary cluster of objects, rather than just single objects. A new statistical specification will be needed to model the clustering possibilities and signaling values.

**Figure 5.**
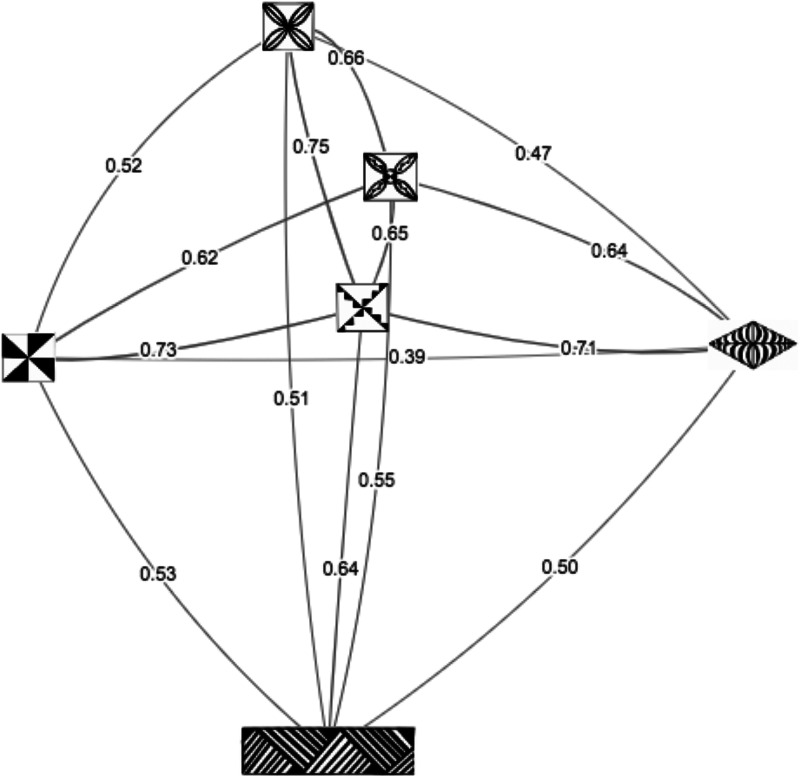
Network showing the relationship among motifs in the classification tasks in the Tongan sample. The spatial positions of nodes was determined through multidimensional scaling using a matrix describing the probability that two motifs were paired across triads, labeled along the edges between nodes.

The ethos of the future work outlined above may follow the sizable literature and data collection effort on semantic representations (Nelson, McEvoy, & Schreiber, 2004). In this literature, building an accurate measure of word meaning has involved competing data collection and statistical methods evaluated against a separate external measure of word meaning. Word associations and the analysis of naturalistic text corpora, as two prominent methods, have yielded varying predictive power with the former often favored (De Deyne, Navarro, Perfors, Brysbaert, & Storms, 2019). One key finding is that the analysis of a semantic network that incorporates indirect relations outperforms the sole use of pairwise associations. It is likely that future theoretical and empirical work on signaling values will incorporate the association structure between objects more explicitly (Figure 5).

## Conclusion

Once the value of objects can be measured then general discussions of group signaling can be transformed into an object-centered discussion that relates object signaling values to theoretical expectations of signaling dynamics, and/or align with ethnographic significance. The analysis of Tongan motifs demonstrates that these possibilities are in reach, relating signaling values to history and contemporary forces of cultural change. Using this method more complex predictions may be addressed relating nested or evolving group boundaries (Wimmer, 2013), demographic forces (McElreath *et al.*, 2003) and covert signaling (Smaldino, Flamson, & McElreath, 2018), revealing key adaptive features of complex human groups.

## Supplementary material

For supplementary material accompanying this paper visit http://dx.doi.org/10.1017/ehs.2020.24. click here to view supplementary material

## Research transparency and reproducibility

All datasets and statistical files are deposited on GitHub: avbell/SignalingValue.
