# Supplementary material for: A measure of social coordination and group signaling in the wild
Source: Evol Hum Sci. 2020 May 14;2:e34. doi: 10.1017/ehs.2020.24 (PMC10427435; doi:10.1017/ehs.2020.24)
Supplement: Supplementary file 1 [file S2513843X20000249sup001.pdf]

Supplementary Material:
A Measure of Social Coordination and Group Signaling in the Wild

Adrian Viliami Bell

May 7, 2020

The condition for object associations via social coordination

Following the description in the main text the fitness functions for individuals associating objects {1,2}, {2,3}, or {1,3} are:

$$
\begin{aligned}
w_{12} &= x_{12}(\delta_1 + \delta_2) + x_{23}\delta_2 + x_{13}\delta_1 \\
w_{23} &= x_{12}\delta_2 + x_{23}(\delta_2 + \delta_3) + x_{13}\delta_3 \\
w_{13} &= x_{12}\delta_1 + x_{23}\delta_3 + x_{13}(\delta_1 + \delta_3)
\end{aligned} \tag{1}
$$

noting $x_{13} = 1 - x_{12} - x_{23}$ and mean fitness is $\bar{w} = x_{12}w_{12} + x_{23}w_{23} + x_{13}w_{13}$. Using a standard viability recursion,

$$x'_{12} = x_{12}w_{12}/\bar{w} \tag{2}$$

$$x'_{23} = x_{23}w_{23}/\bar{w} \tag{3}$$

we can find the difference equation $\Delta x_{ij} = x'_{ij} - x_{ij}$, which we use to find the condition of increase ($\Delta x_{ij} > 0$) yielding result Eq. (1) in the text. Simulation of this system yielded Fig. 1, a ternary plot of the dynamics. See the supporting R code for details, deposited with the author's GitHub depository: avbell/SignalingValue.

Statistical model translated from an evolutionary dynamic

From Table 1 in the main text we constructed a systems of equations describing the evolution of associations. See the Mathematica code below and the R code hosted at the GitHub link above.

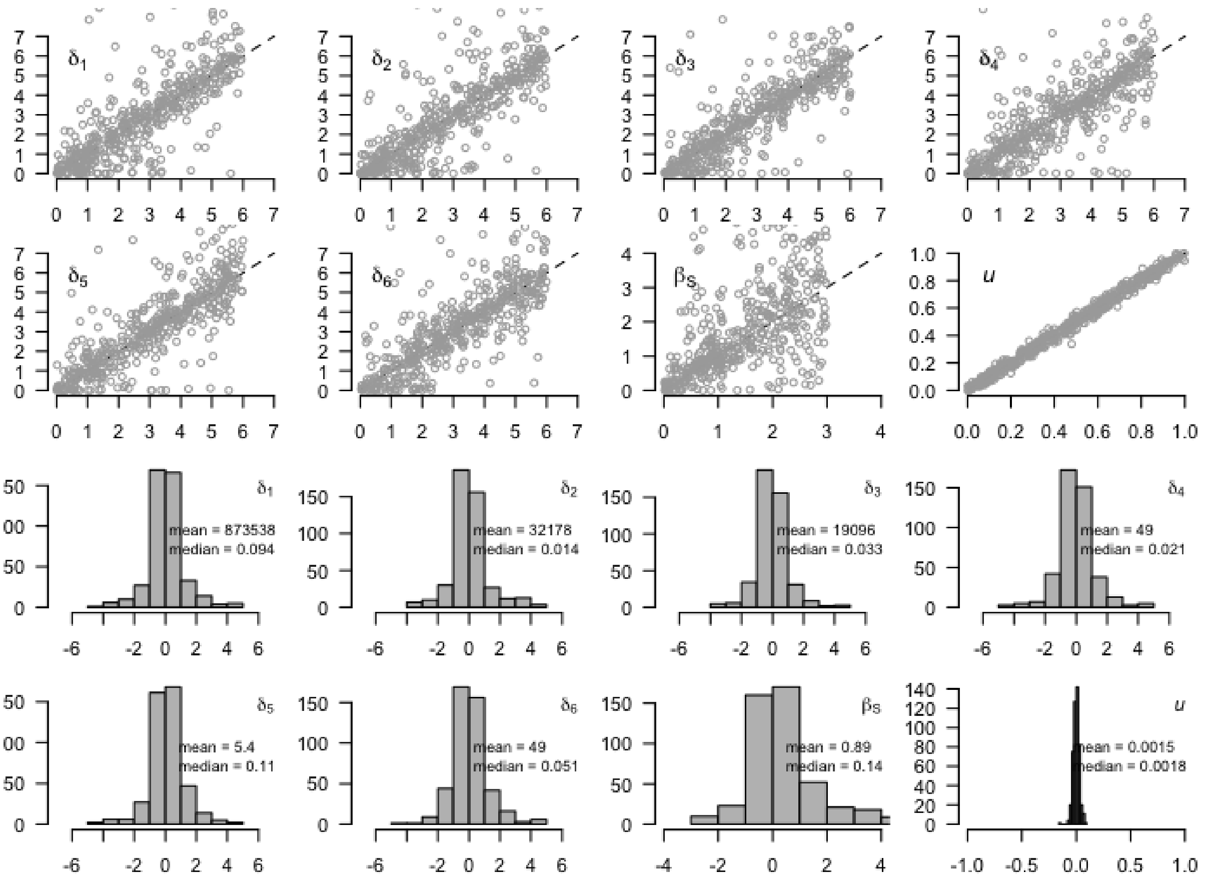

Supplementary Figure S1: Test of parameter estimation from simulated data. For each simulated data set, 100 observations were generated for a 6-object 20-triad classification task. Then maximum likelihood estimates were found using Nelder-Mead optimization with initial parameter values perturbed away from generating values. This was done 1000 times. The scatter plots display estimates (vertical axis) with data-generating parameter values (horizontal axis). Not all points are shown. Dashed line is a guide showing when values are equivalent. The bottom set are histograms of differences between estimated parameters and data-generating parameter values. Mean and median are reported given rare but influential outliers that are not shown on both the scatter plots and histograms.

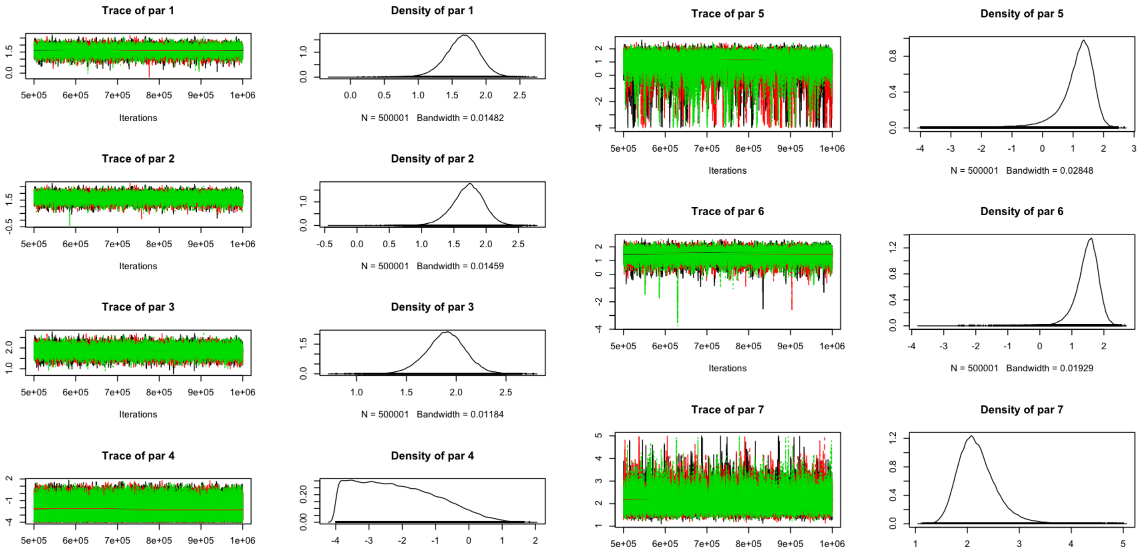

Supplementary Figure S2: Samples of parameters $\delta_1 \ldots \delta_6$, respectively labeled as par1 – par 6, and u (par 7) using the Markov Chain Monte Carlo sampling of the likelihood. Shown here are the second half of one million samples, before transformation, used to calculate the estimates and intervals. The samples for δ_4 corresponding to the motif *amoamokofe* are near zero and the interval borders values that are effectively zero. The transformation for δ_i is through the exponential function and the parameter u is transformed through the inverse-logit function.

Evolution of Symbolic Complexity and Association

Fitness functions

In[40]:=
```
Quit[]
```

In[1]:=
```
w12 := x12 (d1 + d2) + x23 d2 + x13 d1
w23 := x12 d2 + x23 (d2 + d3) + x13 d3
w13 := x12 d1 + x23 d3 + x13 (d1 + d3)
```

In[4]:=
```
wav := x12 w12 + x23 w23 + x13 w13
```

In[5]:=
```
x12p := x12 w12 / wav
x23p := x23 w23 / wav
x13p := x13 w13 / wav
```

In[8]:=
```
dx12  := x12p - x12
dx23 := x23p - x23
x13  := 1 - x12 - x23
```

In[11]:=
```
FullSimplify[dx12]
FullSimplify[dx23]
```

Out[11]= $-\left(\left(x12\left(d3\,(-1+x12)^2+d1\,(-1+x23)\,x23+d2\,(-1+x12+x23)\,(x12+x23)\right)\right)\Big/\left(d3\,(-1+x12)^2+d1\,(-1+x23)^2+d2\,(x12+x23)^2\right)\right)$

Out[12]= $\left(x23\left(-d3\,(-1+x12)\,x12-d1\,(-1+x23)^2-d2\,(-1+x12+x23)\,(x12+x23)\right)\right)\Big/\left(d3\,(-1+x12)^2+d1\,(-1+x23)^2+d2\,(x12+x23)^2\right)$

when dx12>0

In[13]:=
```
FullSimplify[Reduce[dx12 > 0 && d1 > 0 && d2 > 0 && 0 < x12 < 1 && 0 < x23 < 1 ]]
```

Out[13]= $0<x23<1\ \&\&\ 0<x12<1\ \&\&\ d2>0\ \&\&\ d1>0\ \&\&$
$$-\frac{d1\,(-1+x23)^2+d2\,(x12+x23)^2}{(-1+x12)^2}<d3<-\frac{d1\,(-1+x23)\,x23+d2\,(-1+x12+x23)\,(x12+x23)}{(-1+x12)^2}$$

this inequality rearranged is equation [1] in the paper; find equilibrium of system

In[14]:=
```
eq = FullSimplify[Solve[{dx12 == 0, dx23 == 0}, {x12, x23}]]
```

Out[14]= $\left\{\{x12\to 0,\ x23\to 1\},\ \left\{x12\to 0,\ x23\to\frac{d1}{d1+d2}\right\},\ \left\{x12\to\frac{d3}{d1+d3},\ x23\to\frac{d1}{d1+d3}\right\},\right.$
$\left\{x12\to 1-\frac{2\,d1\,d2}{d2\,d3+d1\,(d2+d3)},\ x23\to\frac{-d2\,d3+d1\,(d2+d3)}{d2\,d3+d1\,(d2+d3)}\right\},$
$\left.\{x12\to 0,\ x23\to 0\},\ \{x12\to 1,\ x23\to 0\},\ \left\{x12\to\frac{d3}{d2+d3},\ x23\to 0\right\}\right\}$

given that payoffs depend on coordination, the trivial solutions are likely stable; use simulation to find

stability of equilibria; see R code

Statistical Model of Signal Association

In[16]:=
```
Quit[]
```

Model derived from Table 1 using inverse logit function

check that interaction probabilities add to one

In[1]:=
```
Simplify[x12^2 + x23^2 + x13^2 + 2 x12 x23 + 2 x12 x13 + 2 x23 x13]
```

Out[1]=
```
(x12 + x13 + x23)^2
```

In[2]:=
```
H[x_] := Exp[x] / (1 + Exp[x])
```

In[3]:=
```
x12t := 2 x12 x23 (1/2) H[Bs d2] + 2 x12 x13 (1/2) H[Bs d1] +
   2 x23 x13 (1 - H[Bs d3]) + x12^2 (1 - (1/2) H[Bs (d1 - d2)] - (1/2) H[Bs (d2 - d1)]) +
   x23^2 (1/2) H[Bs (d2 - d3)] + x13^2 (1/2) H[Bs (d1 - d3)]
x13t := 2 x12 x23 (1 - H[Bs d2]) + 2 x12 x13 (1/2) H[Bs d1] + 2 x23 x13 (1/2) H[Bs d3] +
   x12^2 (1/2) H[Bs (d1 - d2)] + x23^2 (1/2) H[Bs (d3 - d2)] +
   x13^2 (1 - (1/2) H[Bs (d1 - d3)] - (1/2) H[Bs (d3 - d1)])
x23t := 2 x12 x23 (1/2) H[Bs d2] + 2 x12 x13 (1 - H[Bs d1]) +
   2 x23 x13 (1/2) H[Bs d3] + x12^2 (1/2) H[Bs (d2 - d1)] +
   x23^2 (1 - (1/2) H[Bs (d2 - d3)] - (1/2) H[Bs (d3 - d2)]) + x13^2 (1/2) H[Bs (d3 - d1)]
```

adds to one (awesome)

In[6]:=
```
FullSimplify[x12t + x13t + x23t]
```

Out[6]=
```
(x12 + x13 + x23)^2
```

While simulations (see R code) will explore the evolutionary dynamics of the model, lets take some looks using calculus and algebra

What influences the direction of change? Derivative of one of the difference equations

In[7]:=
```
FullSimplify[D[x12t - x12, x12]]
```

Out[7]=

$$\left(-1 + x12 + e^{Bs\,d1}\left(-1 + x12 + x13\right) + e^{Bs\,d2}\left(-1 + x12 + x23\right) + e^{Bs\,(d1+d2)}\left(-1 + x12 + x13 + x23\right)\right) / \left(\left(1 + e^{Bs\,d1}\right)\left(1 + e^{Bs\,d2}\right)\right)$$

Lets put some numbers in to explore graphically

In[8]:=
```
x12t - x12 /. {d1 -> 1, d2 -> 1, d3 → 1, Bs → 0.1, x13 → 0.5,  x23 → 1 - x12 - 0.5}
```

Out[8]=

$$0.0625 + 0.475021\,(0.5 - x12) + 0.25\,(0.5 - x12)^2 - 0.73751\,x12 + 0.524979\,(0.5 - x12)\,x12 + 0.5\,x12^2$$

In[9]:=
```
Plot[%, {x12, 0, 0.5}]
```

Out[9]=

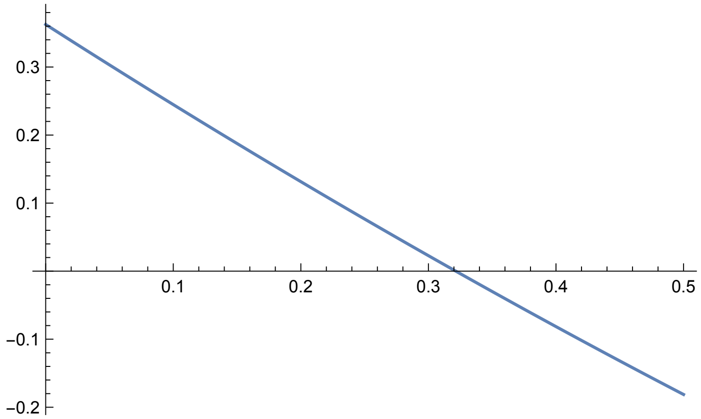

The plot above suggests that, all else equal, we see some frequency dependence, as expected with a model based on coordination

Can we find a closed solution to the stationary states? Likely not, lets try below.

In[10]:=
```
x23 := 1 - x12 - x13
```

In[11]:=
```
Solve[{x12t == x12, x13t == x13}, {x12, x13}]
```

Out[11]=
```
$Aborted
```

the effects of the two-object coordination benefits rely on individual coordination benefits d1 and d2, simple

In[12]:=
```
D[x12t - x12, d1]
```

Out[12]=

$$\left(\frac{\text{Bs}\, e^{2\,\text{Bs}\,(\text{d1}-\text{d2})}}{2\left(1+e^{\text{Bs}\,(\text{d1}-\text{d2})}\right)^2} - \frac{\text{Bs}\, e^{\text{Bs}\,(\text{d1}-\text{d2})}}{2\left(1+e^{\text{Bs}\,(\text{d1}-\text{d2})}\right)} - \frac{\text{Bs}\, e^{2\,\text{Bs}\,(-\text{d1}+\text{d2})}}{2\left(1+e^{\text{Bs}\,(-\text{d1}+\text{d2})}\right)^2} + \frac{\text{Bs}\, e^{\text{Bs}\,(-\text{d1}+\text{d2})}}{2\left(1+e^{\text{Bs}\,(-\text{d1}+\text{d2})}\right)}\right) \text{x12}^2 - \frac{\text{Bs}\, e^{2\,\text{Bs}\,\text{d1}}\,\text{x12}\,\text{x13}}{\left(1+e^{\text{Bs}\,\text{d1}}\right)^2} + \frac{\text{Bs}\, e^{\text{Bs}\,\text{d1}}\,\text{x12}\,\text{x13}}{1+e^{\text{Bs}\,\text{d1}}} - \frac{\text{Bs}\, e^{2\,\text{Bs}\,(\text{d1}-\text{d3})}\,\text{x13}^2}{2\left(1+e^{\text{Bs}\,(\text{d1}-\text{d3})}\right)^2} + \frac{\text{Bs}\, e^{\text{Bs}\,(\text{d1}-\text{d3})}\,\text{x13}^2}{2\left(1+e^{\text{Bs}\,(\text{d1}-\text{d3})}\right)}$$

Use simulation to visualize the dynamics of the system; see R code
